# An unappreciated cell survival-independent role for BAFF initiating chronic lymphocytic leukemia

**DOI:** 10.3389/fimmu.2024.1345515

**Published:** 2024-02-26

**Authors:** Md Ashik Ullah, Beatriz Garcillán, Eden Whitlock, William A. Figgett, Simona Infantino, Mahya Eslami, SiLing Yang, M. Arifur Rahman, Yong H. Sheng, Nicholas Weber, Pascal Schneider, Constantine S. Tam, Fabienne Mackay

**Affiliations:** ^1^Queensland Institute of Medical Research (QIMR) Berghofer Medical Research Institute, Cancer Program, Herston, QLD, Australia; ^2^The Department of Microbiology and Immunology, School of Biomedical Sciences, Faculty of Medicine, Dentistry and Health Sciences, University of Melbourne, Parkville, VIC, Australia; ^3^Garvan Institute of Medical Research, Kinghorn Centre for Clinical Genomics, Darlinghurst, NSW, Australia; ^4^Department of Immunobiology, University of Lausanne, Epalinges, Switzerland; ^5^Department of Oncology and Children’s Research Centre, University Children’s Hospital Zürich, Zürich, Switzerland; ^6^Cancer Care Services, Royal Brisbane and Women’s Hospital, Herston, QLD, Australia; ^7^Department of Haematology, Alfred Hospital, Melbourne, VIC, Australia; ^8^Department of Haematology, Monash University, Melbourne, VIC, Australia; ^9^The Department of Immunology and Pathology, Monash University, VIC, Australia; ^10^Faculty of Medicine, University of Queensland, Brisbane, QLD, Australia

**Keywords:** chronic lymphocytic leukemia, BAFF, APRIL, TCL1-Tg mice model, leukemia initiation, leukemia dissemination

## Abstract

**Background:**

Chronic Lymphocytic Leukemia (CLL) is characterized by the expansion of CD19^+^ CD5^+^ B cells but its origin remains debated. Mutated CLL may originate from post-germinal center B cells and unmutated CLL from CD5^+^ mature B cell precursors. Irrespective of precursor types, events initiating CLL remain unknown. The cytokines BAFF and APRIL each play a significant role in CLL cell survival and accumulation, but their involvement in disease initiation remains unclear.

**Methods:**

We generated novel CLL models lacking BAFF or APRIL. *In vivo* experiments were conducted to explore the impact of BAFF or APRIL loss on leukemia initiation, progression, and dissemination. Additionally, RNA-seq and quantitative real-time PCR were performed to unveil the transcriptomic signature influenced by BAFF in CLL. The direct role of BAFF in controlling the expression of tumor-promoting genes was further assessed in patient-derived primary CLL cells *ex-vivo*.

**Results:**

Our findings demonstrate a crucial role for BAFF, but not APRIL, in the initiation and dissemination of CLL cells. In the absence of BAFF or its receptor BAFF-R, the TCL1 transgene only increases CLL cell numbers in the peritoneal cavity, without dissemination into the periphery. While BAFF binding to BAFF-R is dispensable for peritoneal CLL cell survival, it is necessary to activate a tumor-promoting gene program, potentially linked to CLL initiation and progression. This direct role of BAFF in controlling the expression of tumor-promoting genes was confirmed in patient-derived primary CLL cells ex-vivo.

**Conclusions:**

Our study, involving both mouse and human CLL cells, suggests that BAFF might initiate CLL through mechanisms independent of cell survival. Combining current CLL therapies with BAFF inhibition could offer a dual benefit by reducing peripheral tumor burden and suppressing transformed CLL cell output.

## Introduction

Chronic Lymphocytic Leukemia (CLL) is the most prevalent form of adult leukemia in the developed world, typically affecting patients later in life (1). CLL is characterized by the accumulation of monoclonal neoplastic activated mature CD5^+^ B cells (CLL B cells) initially in the blood and in primary and secondary lymphoid organs as the disease progresses (1, 2). CLL is a clinically heterogeneous disease, with some patients developing an aggressive form of CLL requiring treatment and others developing an indolent form not treated but monitored for progression. CLL remains incurable despite significant advances in the management of patients. CLL cells express either mutated or unmutated immunoglobulin variable regions (IgV) within their B cell receptors (BCR), with unmutated status generally correlating with poorer prognosis (2). The cell-of-origin from which CLL cells derive remains elusive; transcriptome analysis suggests that unmutated CLL cells derive from CD5^+^ mature B cells, whereas mutated CLL cells derive from post-germinal center CD27^+^ B cells (3). The tumor microenvironment (TME) provides pro-survival signals and protects tumor cells from spontaneous and drug-induced apoptosis. CLL cells shape their microenvironment not only to support their survival and proliferation but to escape anti-tumor immune surveillance (4).

B cell-activating factor (BAFF) and a proliferation-inducing ligand (APRIL) of the tumor necrosis factor (TNF) family are produced by CLL cells and cells in the TME (5–7). Both ligands contribute to CLL cell survival (5, 6). Intracellular expression of BAFF or APRIL in CLL cells correlates with poorer prognosis (8). However, only levels of APRIL are elevated in the serum of patients with CLL compared to healthy subjects (8–10). Elevated APRIL and low BAFF serum levels are associated with poorer prognosis (9). Over-expression of human APRIL in mice leads to the expansion of CD5^+^ neoplastic B cells and the development of a disease that resembles progressive CLL (11). High expression of the oncogenic T cell leukemia 1 (TCL1) gene is a marker of progressive CLL with poor prognosis (12), and mice overexpressing human TCL1 gene under a B cell-specific promoter (Eµ-TCL1-Tg mice, referred to as TCL1-Tg mice) develop progressive CLL (13). Critically, CLL progression in this model is accelerated when either human APRIL (14) or mouse BAFF (15) is overexpressed. BAFF promotes the survival of CLL cells *in vivo* and *in vitro*, with a lower proportion of apoptotic CLL cells observed when BAFF levels are elevated (15). However, the exact role of APRIL in CLL cell survival remains unclear, with an independent study failing to see any APRIL-mediated survival of human CLL cells *in vitro* (7).

Both BAFF and APRIL bind to the transmembrane activator and cyclophilin ligand interactor (TACI) and B cell maturation antigen (BCMA) receptors (16). BAFF, but not APRIL, binds to the BAFF receptor (BAFF-R) (16). In TCL1-Tg mice overexpressing human APRIL, the development of CLL depends on TACI but not BCMA signaling (14). Decreased levels of BAFF in CLL correlate with greater levels of circulating soluble TACI (sTACI) which correlate with worse outcomes in patients (17).

Collectively, the data support a potentially vital role for the APRIL-TACI axis in the survival and progression of CLL; however, the exact contribution of BAFF or APRIL in the initiation of CLL prior to progression *in vivo* remains untested. Here, we show that similar to patients with CLL, serum levels of APRIL are elevated and BAFF levels decreased in the TCL1-Tg model of CLL. TCL1-Tg mice lacking BAFF, but not APRIL, were protected against CLL development with fewer residual CLL cells sequestered in the peritoneal cavity. Our results demonstrate that BAFF is required for the dissemination of peritoneal CLL cells but not their survival during initiation of CLL.

## Materials and methods

### Mice

Animal work adhered to approved protocols from The University of Melbourne and QIMR Berghofer Medical Research Institute Animal Care and Ethics Committees. The study utilized TCL1-Tg mice on a C57BL/6 background, which were kindly donated by Prof. David Huang (13). WT mice (C57BL/6) were sourced from the Animal Resources Centre (ARC, Australia); APRIL^-/-^ mice were sourced from the knockout mouse project (KOMP) repository, and the phenotype of BAFF^-/-^ and BAFF-R^-/-^ mice has been described previously (18, 19). BAFF^-/-^, BAFF-R^-/-^ and APRIL^-/-^ mice were crossed onto TCL1-Tg mice to generate novel models of CLL lacking BAFF, BAFF-R, APRIL or both BAFF and APRIL, respectively. Mice were bled monthly to monitor leukemia development starting at 2 months of age. The ethical endpoint was reached when CLL cell counts exceeded 1x10^4^ cells/µl blood. Mice were kept in pathogen-free conditions and monitored weekly. After euthanasia, peritoneal cells were collected by washing the peritoneal cavity with 10 ml of cold FACS buffer. In adoptive transfer experiments, 1.3 x 10^6^ peritoneal CLL cells from TCL1-Tg mice were injected i.p. or i.v. into recipient mice. In separate experiments, 7 to 8 months old TCL1-Tg and TCL1-Tg BAFF^-/-^ mice were injected i.p. with anti-APRIL (Centotto-1, Adipogen, Lausen, Switzerland) or a control antibody once a week for 8 weeks at a dose of 2 mg/kg (20). Seven days after the final treatment, blood and immune organs (spleen, bone marrow, lymph nodes, peritoneal lavage, and omentum) were collected for analysis. Single-cell suspensions were prepared for flow cytometry, cell culture, and gene expression analysis.

### ELISA

Commercially available ELISA kits were used to measure serum levels of mouse BAFF (Quantikine kit; R&D Systems, MN, USA), mouse APRIL (Antibodies-online Inc., PA, USA) and soluble mouse TACI (sTACI; R&D Systems, MN, USA).

### CLL cell culture and gene expression analysis

FACS-sorted purified peritoneal CLL cells from TCL1-Tg and TCL1-Tg BAFF^-/-^ mice or magnetic-sorted human CLL cells were stimulated with 50 ng/ml of recombinant mouse or human BAFF, respectively. Gene expression was analyzed using RT-qPCR. RNA libraries were prepared from mouse peritoneal cells and subjected to bulk RNA-sequencing.

For original data, please contact fabienne.mackay@qimrberghofer.edu.au. RNA-sequencing data is deposited in Gene Expression Omnibus. Additional methods are included in supplemental materials.

### Statistics

Data are presented as mean ± standard error of the mean (SEM). Data were analyzed using Mann-Whitney t-test, paired t-test and one-way or two-way ANOVA with Bonferroni ***post hoc*** test as appropriate. Survival and CLL incidence were evaluated using Kaplan-Meier methods and different groups were calculated using the Log-rank (Mantel-Cox) test. All the analysis was performed using Prism version 9 (GraphPad Software, CA, USA). The statistically significant results were annotated with *P < 0.05, **P < 0.01, ***P < 0.001, ****P < 0.0001 and ns for not significant.

### Study approval

All studies involving human biospecimens were conducted following approval by the Royal Brisbane & Women’s Hospital Human Research Ethics Committee. The written informed consent was received prior to participation. All animal studies were conducted in accordance with protocols reviewed and approved by the Queensland Institute of Medical Research (QIMR) Berghofer Medical Research Institute Animal Research Ethics Committee.

## Results

### Levels of BAFF and APRIL ligands and receptors in TCL1-Tg mice parallel those described in human CLL

**Figure 1:**
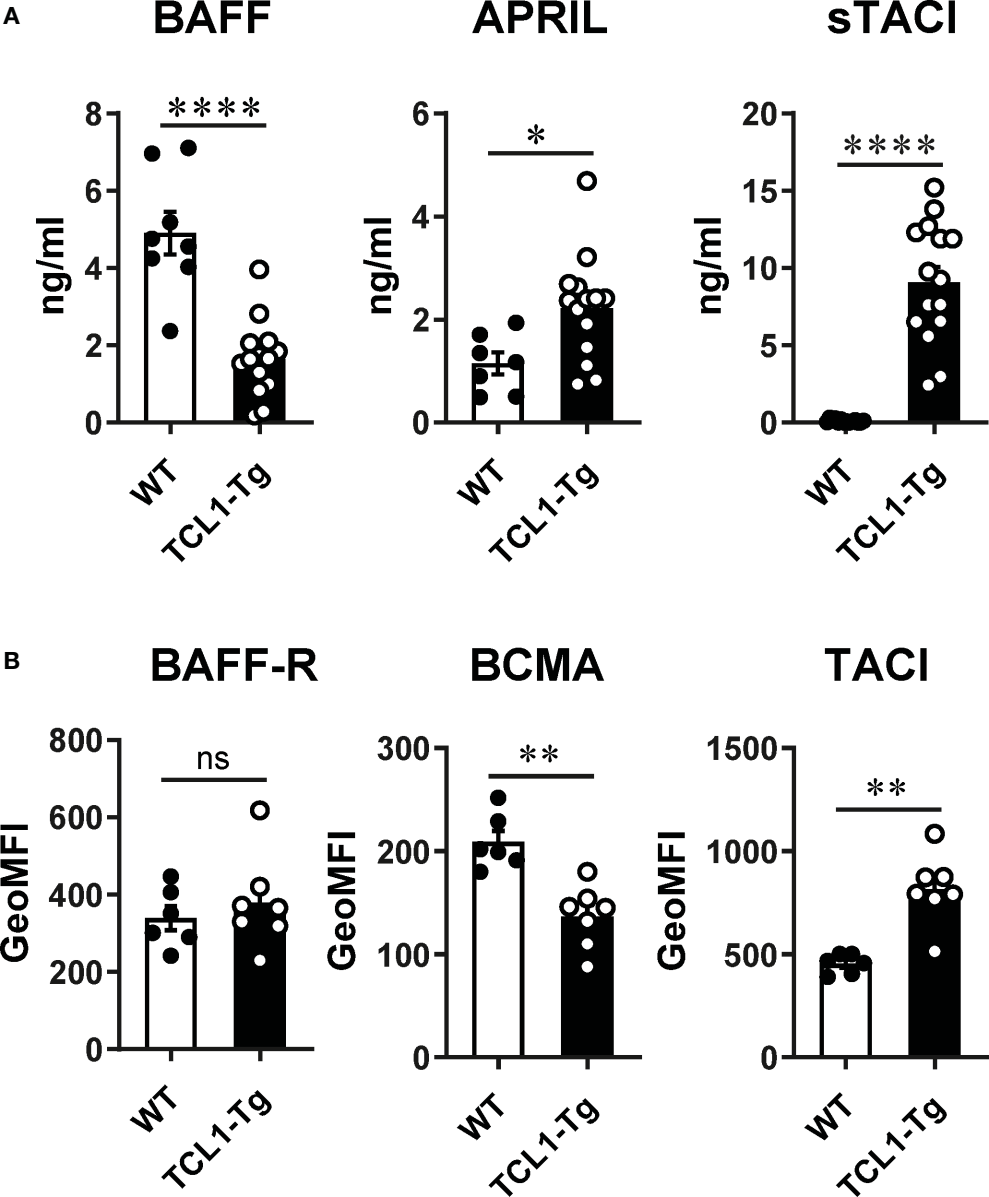
Levels of BAFF and APRIL ligands and receptors on normal B1a B cells and CLL cells. **(A)** Circulating BAFF, APRIL and sTACI levels were measured by ELISA from TCL1-Tg mice and age-matched WT controls. **(B)** Surface expression of BAFF-R, BCMA and TACI was determined by flow cytometry on blood CD19^+^CD5^+^ cells from TCL1-Tg mice and age-matched WT controls (B1a B cells). The data is presented as mean ± SEM. Mann-Whitney t-tests were used for comparisons. Not significant (ns), *P < 0.05, **P < 0.01, ***P < 0.001, ****P < 0.0001.

We compared the expression profile of BAFF, APRIL and their receptors on CLL cells in the TCL1-Tg CLL mouse model to that described for human CLL (7–10, 21). Similar to independent observations in separate cohorts of CLL patients (7–10), serum levels of BAFF were reduced while that of APRIL and sTACI were significantly increased in comparison to WT control mice (**Figure 1A**). Similar to human CLL cells (21, 22), CLL cells in TCL1-Tg mice expressed BAFF-R, BCMA and TACI with notably higher TACI and lower BCMA cell surface expression relative to CD5^+^CD19^+^ B1 B cells in WT mice (**Figure 1B**). The levels of BAFF-R expression were comparable between WT CD5^+^ B cells and TCL1-Tg CLL cells (**Figure 1B**). The expression profiles of BAFF, APRIL, their respective surface receptors and sTACI in TCL1-Tg mice paralled that descrived in human CLL, supporting the use of this mouse model to explore the role of BAFF and APRIL in CLL initiation.

### BAFF, but not APRIL, is required for CLL initiation and progression in TCL1-Tg mice

**Figure 2:**
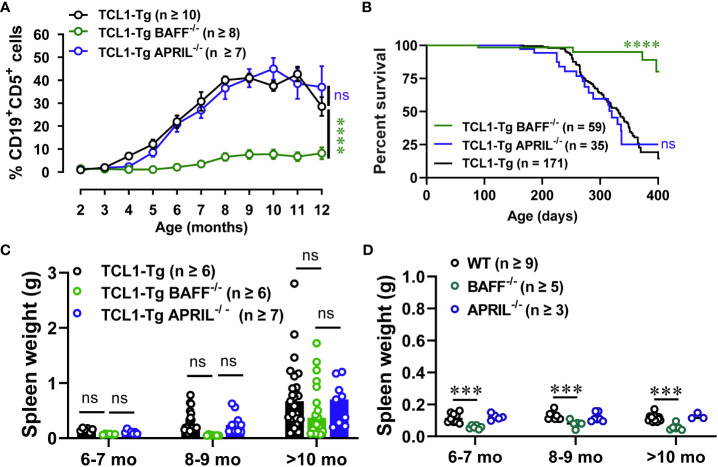
TCL1-Tg BAFF^-/-^, but not TCL1-Tg APRIL^-/-^, mice are protected from CLL. **(A)** Percentages of CD19^+^CD5^+^ cells (CD45.2^+^ live gate) in the blood of 2 to 12 months old TCL1-Tg, TCL1-Tg BAFF^-/-^ and TCL1-Tg APRIL^-/-^ mice determined by flow cytometry. **(B)** Kaplan-Meier curve depicting overall survival from birth to ethical endpoint of TCL1-Tg (median survival = 332 days), TCL1-Tg BAFF^-/-^ (median survival = 434 days) and TCL1-Tg APRIL^-/-^ (median survival = 320 days). **(C)** Weights of spleens from TCL1-Tg, TCL1-Tg BAFF^-/-^ and TCL1-Tg APRIL^-/-^ mice, and **(D)** WT, BAFF^-/-^ and APRIL^-/-^ age 6 to >10 months as indicated. Data presented as mean values ± SEM. A, C Differences between the groups were analyzed using two-way ANOVA with Bonferroni *post hoc* test. B Survival data were compared using the Log-rank (Mantel-Cox) test. Not significant (ns), ***P < 0.001, ****P < 0.0001. The number of mice varies with endpoints at different time points.

To determine the relative contribution of BAFF and APRIL in CLL initiation and progression in TCL1-Tg mice, we generated TCL1-Tg mice lacking BAFF or APRIL and followed spontaneous disease progression over 12 months. Interestingly, BAFF but not APRIL was critical for CLL initiation and progression, as TCL1-Tg BAFF^-/-^ mice had reduced frequency and absolute numbers of CD5^+^CD19^+^ CLL cells in the blood (**Figure 2A**; **Supplementary Figure 1A**). In contrast, TCL1-Tg APRIL^-/-^ mice developed CLL similar to that in control TCL1-Tg mice. CLL incidence was also similar in the absence or presence of APRIL but significantly reduced in the absence of BAFF (**Supplementary Figure 1B**). TCL1-Tg mice lacking BAFF survived to an older age compared to TCL1-Tg and TCL1-Tg APRIL^-/-^ mice (**Figure 2B**). TCL1-Tg BAFF^-/-^ mice exhibited a decreasing trend in spleen weight up to 9 months of age compared to both TCL1-Tg and TCL1-Tg APRIL^-/-^ mice (**Figure 2C**), along with spleen sizes similar to those observed in BAFF^-/-^ control mouse groups (**Figure 2D**). Notably, an increase in spleen size was observed only in TCL1-Tg BAFF^-/-^ mice aged over 10 months (**Figure 2C**), an unrelated change not linked to increased numbers of CLL cells in these mice which remained low irrespective of age (**Figure 2A**). Collectively, our results suggest that initiation and development of progressive CLL in TCL1-Tg mice are highly dependent on BAFF but not APRIL.

### BAFF, but not APRIL, is required for peripheral CLL development but not CLL survival and expansion in the peritoneal cavity

**Figure 3:**
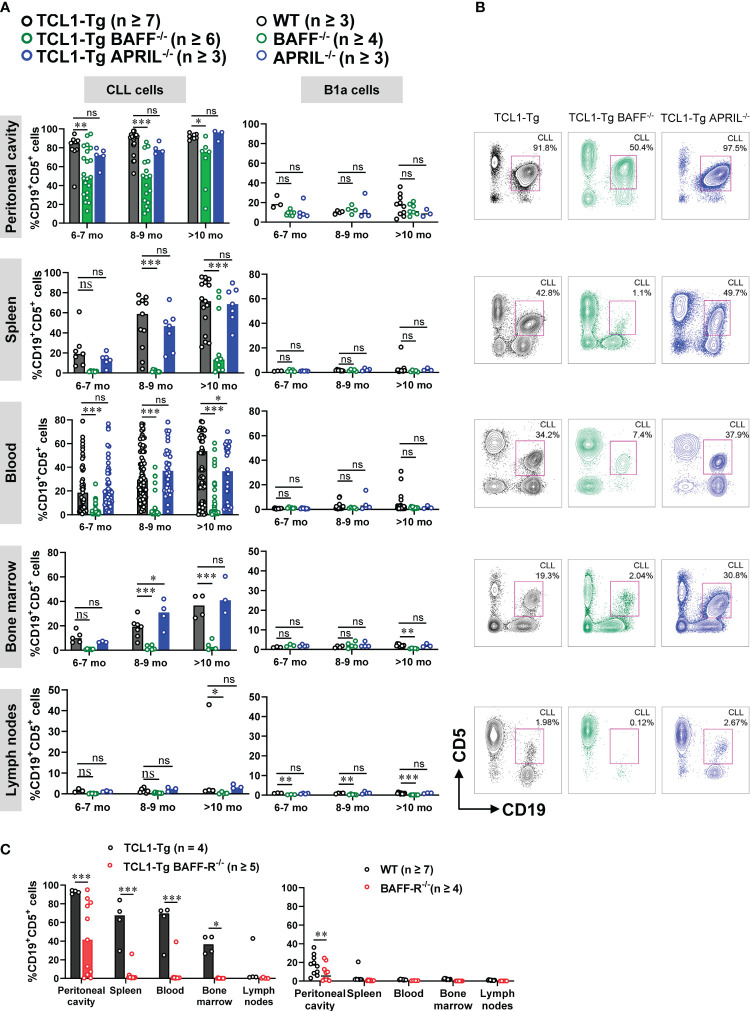
BAFF, but not APRIL, is required for CLL development. **(A)** Percentages of CD19^+^CD5^+^ cells (CD45.2^+^ live gate) in the peritoneal cavity, spleen, blood, bone marrow and lymph nodes of 6- to 10-months old TCL1-Tg, TCL1-Tg BAFF^-/-^, TCL1-Tg APRIL^-/-^, WT, BAFF^-/-^ and APRIL^-/-^ mice, as indicated. **(B)** Representative flow cytometric plots with gated CD19^+^CD5^+^ cells from TCL1-Tg, TCL1-Tg BAFF^-/-^ and TCL1-Tg APRIL^-/-^ mice at 8-9 months of age in (from top to bottom) the peritoneal cavity, spleen, blood, bone marrow and lymph nodes, as indicated. **(C)** Percentages of CD19^+^CD5^+^ cells (CD45.2^+^ live gate) in the peritoneal cavity, spleen, blood, bone marrow and lymph nodes of >10 months old TCL1-Tg, TCL1-Tg BAFF-R^-/-^, WT and BAFF-R^-/-^ mice. Statistically significant differences calculated using two-way ANOVA with Bonferroni *post hoc* test. Not significant (ns), *P < 0.05, **P < 0.01, ***P < 0.001. The number of mice varies with endpoints at different time points.

We analyzed the accumulation of CD5^+^CD19^+^ CLL cells over time in the following immune compartments: spleen, blood, bone marrow, lymph nodes and peritoneal cavity of TCL1-Tg and TCL1-Tg mice lacking BAFF or APRIL. Normal CD19^+^CD5^+^ cells (B1a cells) were analyzed in corresponding control genotypes; WT, BAFF^-/-^ and APRIL^-/-^ (**Figure 3A, B**; **Supplementary Figure 2A**). A similar accumulation of CLL cells was observed in all compartments in TCL1-Tg and TCL1-Tg APRIL^-/-^ mice, with an exception in older TCL1-Tg APRIL^-/-^ mice having fewer CLL cells in the blood at 10 months but more in the bone marrow at age 8-9 months (**Figure 3A**). In contrast, the proportion of CLL cells in the blood, spleen and bone marrow of TCL1-Tg BAFF^-/-^ mice was significantly reduced compared to TCL1-Tg mice (**Figure 3A**). Interestingly, TCL1-Tg BAFF^-/-^ produced higher numbers of peritoneal CD19^+^CD5^+^ cells when compared to peritoneal CD19^+^CD5^+^ B1a B cell numbers in the BAFF^-/-^ control mice. However, peritoneal CD19^+^CD5^+^ cell numbers in TCL1-Tg BAFF^-/-^ are lower than those observed in TCL1-Tg controls or TCL1-Tg-APRIL^-/-^ mice (**Figure 3A, B**; **Supplementary Figure 2A**).

We also investigated whether BAFF signaling via BAFF-R is critical for the initiation of CLL development. Similar to CLL in TCL1-Tg BAFF^-/-^ mice, TCL1-Tg BAFF-R^-/-^ mice also had significantly reduced CLL burden (**Figure 3C**; **Supplementary Figure 2B**). Similarly, the proportion of CLL cells was also elevated in the peritoneal cavity of TCL1-Tg BAFF-R^-/-^ mice (**Figure 3C**). In conclusion, TCL1-Tg mice lacking BAFF or BAFF-R are protected against the development of CLL, with lower levels of residual CLL disease detected in the peritoneal cavity.

We also examined other immune compartments. Notably, the proportions of splenic regulatory Foxp3^+^ CD25^+^ CD4 T cells (Treg) cells are similarly elevated in the TCL1-Tg and TCL1-Tg APRIL^-/-^ mice, but significantly reduced in TCL1-Tg BAFF^-/-^ (**Supplementary Figure 3A**). The role of T cells in CLL is still debated (23), but Tregs may contribute to inhibiting tumor immunity and promoting cancer progression. Here we demonstrate that BAFF is required to maintain high numbers of Tregs in TCL1-Tg mice (**Supplementary Figure 3A**).

### CLL cells are sequestered in the peritoneal cavity of TCL1-Tg BAFF^-/-^ mice

**Figure 4:**
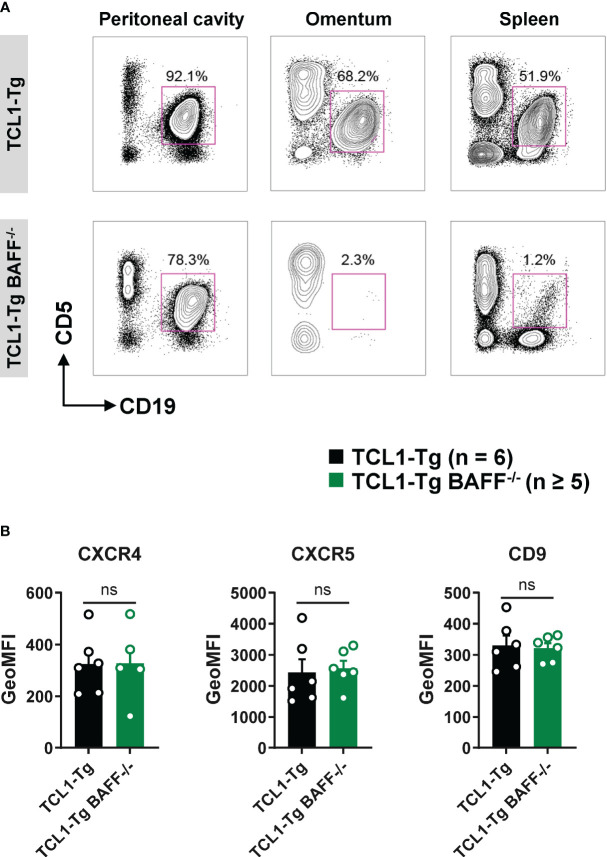
CLL cells are sequestered in the peritoneal cavity of TCL1-Tg BAFF^-/-^ mice. **(A)** Representative flow cytometry plots depicting gated CD19^+^CD5^+^ B cells (CD45.2^+^ live gate) in the peritoneal cavity, omentum and spleen of 8 months old TCL1-Tg and TCL1-Tg BAFF^-/-^ mice as indicated. **(B)** Surface expression of CXCR4, CXCR5 and CD9 on peritoneal CD19^+^CD5^+^ B cells determined by flow cytometry and expressed as geometric mean fluorescence intensity (GeoMFI). Bar graphs presenting mean values ± SEM, statistically significant differences calculated using Mann-Whitney t-test, not significant (ns).

B1a B cells (CD5^+^ CD19^+^) from the peritoneal compartment are known to recirculate between the peritoneal cavity and the peripheral blood via the omentum in a CXCL13- and CD9-dependent manner (24, 25). Receptors CXCR4 and CXCR5 are essential for CLL migration and survival, and CD9 promotes B1a B cell egress from the peritoneal cavity after toll-like receptor (TLR) stimulation (24, 25). To elucidate the mechanisms responsible for CLL cell retention in the peritoneal cavity of TCL1-Tg BAFF^-/-^ mice, the presence of CLL cells in the omentum was analyzed. CLL cells were present in high frequency in the omentum of TCL1-Tg mice but not that of TCL1-Tg BAFF^-/-^ mice (**Figure 4A**; **Supplementary Figure 3B**). The surface expression of CXCR4, CXCR5 and CD9 was similar on peritoneal cavity CLL cells from TCL1-Tg and TCL1-Tg BAFF^-/-^ mice (**Figure 4B**), ruling out this mechanism as an explanation for CLL sequestration in the peritoneal cavity in the absence of BAFF.

We hypothesized that BAFF and APRIL might play redundant roles in maintaining peritoneal CLL cells, and APRIL might support the survival and retention of CLL cells in the peritoneal cavity of TCL1-Tg BAFF^-/-^ mice. However, i.p. treatment with a neutralizing anti-APRIL antibody did not affect the proportion and absolute numbers of CLL cells in the peritoneal cavity of TCL1-Tg BAFF^-/-^ mice, eliminating APRIL as a significant factor in maintaining the peritoneal CLL compartment in TCL1-Tg BAFF^-/-^ mice (**Supplementary Figure 4A, B**). To validate our findings, we generated TCL1-Tg BAFF^-/-^APRIL^-/-^ double knockout (KO) mice. Similarly, CLL cells failed to develop in the periphery, showing a rate of less than 2% as opposed to 30% in WT controls (**Supplementary Figure 4C**)); However, peritoneal TCL1-Tg BAFF^-/-^ APRIL^-/-^ CLL cells expanded in the peritoneal cavity to what was observed in TCL1-Tg BAFF^-/-^ mice, albeit to a lesser extent than peritoneal CLL cells in TCL1-Tg controls (**Supplementary Figure 4C**; **Figure 3A**). This implies that the loss of APRIL expression in TCL1-Tg BAFF^-/-^ mice had no impact on peritoneal CLL cells and is not responsible for the expansion of these cells. These results parallel what we observed using the anti-APRIL blocking antibody as a treatment (**Supplementary Figure 4A-C**). These observations reinforce the critical role of BAFF, rather than APRIL, in the initiation and progression of CLL in TCL1-Tg mice.

### The BAFF-BAFF-R axis is required for CLL cell dissemination in the periphery but not their survival in the peritoneal cavity

**Figure 5:**
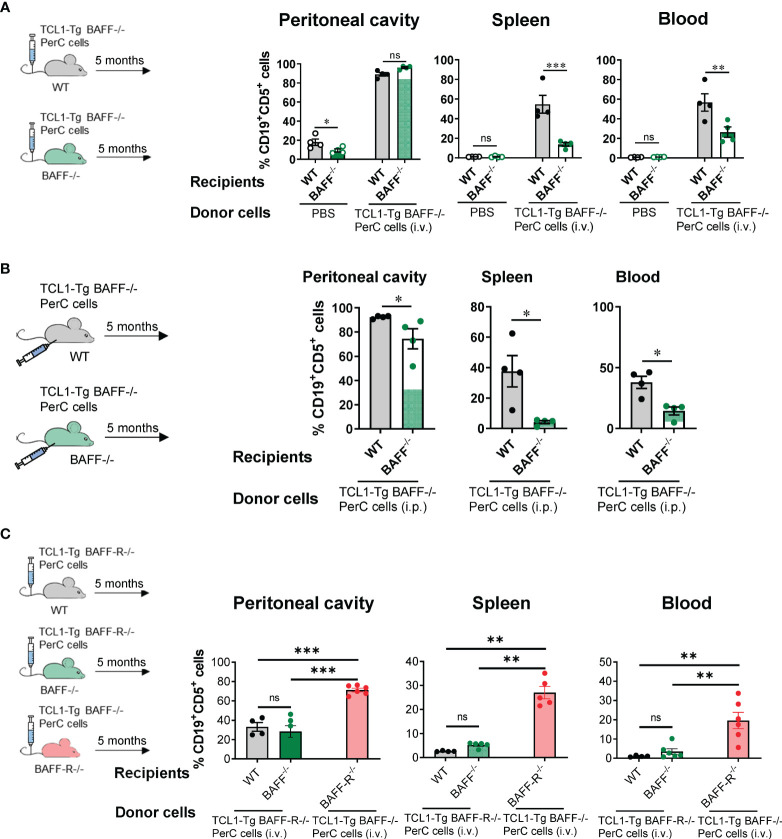
The BAFF-BAFF-R axis is required for CLL cell dissemination but not cell survival in the peritoneal cavity. **(A)** WT or BAFF^-/-^ mice were injected i.v. with 1.3x10^6^ peritoneal cavity (PerC) cells from TCL1-Tg BAFF^-/-^ donor mice. **(B)** WT or BAFF^-/-^ mice were injected i.p. with 1.3x10^6^ PerC cells from TCL1-Tg BAFF^-/-^ donor mice. **(C)** WT or BAFF^-/-^ mice were injected i.v. with 1.3x10^6^ PerC cells from TCL1-Tg BAFF-R^-/-^ donor mice. BAFF-R^-/-^ mice were injected i.v. with PerC cells from TCL1-Tg BAFF^-/-^ donor mice. Respective donor mice were sex-matched and 8 months old. **(A-C)** The percentages of CD19^+^CD5^+^ cells in the peritoneal cavity, spleen and blood (as indicated) were determined by flow cytometry 5 months after injection. Bars represent mean values ± SEM for ≥ 4 mice per group. Statistical analysis was performed using one-way ANOVA with Bonferroni *post hoc* test (A & C) or Mann-Whitney t-test **(B)**. Not significant (ns), *P < 0.05, **P < 0.01, ***P < 0.001.

The possibility is that peritoneal CLL cells from TCL1-Tg BAFF^-/-^ mice might be a residual subgroup distinct from peripheral CLL cells and intrinsically unable to drive CLL and populate other compartments, even when BAFF is present. To investigate, 1.3x10^6^ peritoneal donor CLL cells from TCL1-Tg BAFF^-/-^ mice were intravenously injected into WT or BAFF^-/-^ recipients. Over time, these cells in the blood were tracked by flow cytometry, identified as CD45.2^+^CD5^+^CD19^+^ cells. Five months later, donor TCL1-Tg BAFF^-/-^ CLL cells infiltrated and expanded similarly in the peritoneal cavity of WT or BAFF^-/-^ recipients, as illustrated in **Figure 5A**. Notably, while WT recipients developed CLL, BAFF^-/-^ recipients did not. This suggests that TCL1-Tg BAFF^-/-^ CLL cells are not intrinsically defective and can drive CLL in a BAFF-sufficient environment (**Figure 5A, B**). This suggests that CLL cells may require a local tissue environment with sufficient BAFF levels to disseminate from the peritoneal cavity to the periphery. Additionally, it is also possible that these cells do exit the peritoneal cavity but are unable to thrive in the periphery without BAFF. We also injected TCL1-Tg BAFF^-/-^ peritoneal CLL cells directly into the peritoneal cavity of WT or BAFF^-/-^ mice, and similar results were obtained (**Figure 5B**). These experiments indicate that regardless of the injection route, BAFF-deficient CLL cells can only home, survive and expand in the peritoneal cavity in the absence of BAFF but are fully competent precursors of progressive CLL in WT hosts. These results and lack of CLL cells in the omentum of TCL1-Tg BAFF^-/-^ mice tend to support the notion that BAFF is required for initiation and dissemination of CLL, in addition to its known cell survival requirement in the periphery (**Figure 5B**).

Following this, the requirement of BAFF-R expression on CLL cells for escape from the peritoneal cavity was investigated. When peritoneal TCL1-Tg BAFF-R^-/-^ CLL cells were injected i.v. into WT or BAFF^-/-^ recipient mice, BAFF-R^-/-^ CLL cells were detected in the peritoneal cavity but their proportions were significantly reduced in the spleen and blood of both BAFF^-/-^ and WT recipient mice (**Figure 5C**). Importantly, as BAFF^-/-^ CLL cells are not intrinsically defective (**Figure 5A**), these cells were able to drive CLL in a BAFF-R^-/-^ host producing BAFF, a combination use as CLL development control in **Figure 5C**. Together, our data suggest that CLL cells require BAFF supplied by the host and expression of BAFF-R on the cell surface to drive CLL, thus validating the BAFF-BAFF-R axis as a critical step for the initiation of CLL. These results also indicate that CLL cells survive and expand in the peritoneal cavity in the absence of BAFF production, similar to normal peritoneal B1 B cells (26). BAFF-R expression on CLL cells is needed for these cells to fully egress from the peritoneal cavity to the blood and the spleen, irrespective of the BAFF-R expression status in these tissues (**Figure 5C**).

### BAFF promotes the expression of tumor-promoting genes in CLL cells

**Figure 6:**
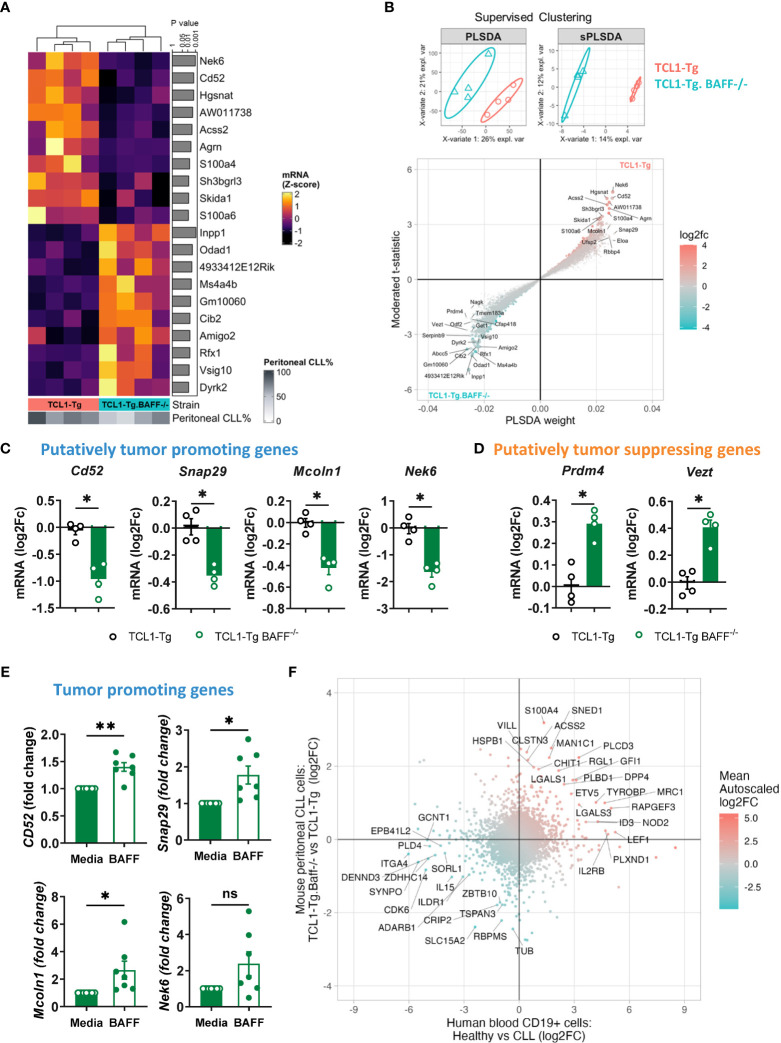
BAFF is required for the expression of tumor-promoting genes in CLL cells. RNA samples from peritoneal CLL cells isolated from TCL1-Tg and TCL1-Tg BAFF^-/-^ mice (n=4/group) were processed for sequencing. **(A)** The top-10 up or down-regulated genes from analysis with limma, ranked by nominal *P*-value were plotted as a heatmap of standardized expression values. **(B)** Supervised clustering by PLSDA and sPLSDA was used to identify additional genes discriminating the two sample groups. To visualize the ranking of genes (from A and B), the moderated t-statistic from limma was plotted against the component weight from PLSDA; symbols represent genes colored by log2 fold-change. **(C, D)** Log2 fold change (FC) of **(C)** putatively tumor-promoting and **(D)** tumor-suppressing genes in TCL1-Tg and TCL1-Tg BAFF^-/-^ CLL cells. Statistical analysis was performed using the Mann-Whitney t-test. n= 4/group, *P < 0.05. **(E)** Tumor-promoting gene expression (as indicated) in purified peritoneal CLL cells of TCL1-Tg BAFF^-/-^ mice stimulated or not for 24 hours *ex-vivo* with recombinant BAFF. Gene expression assessed by qPCR (n=7 mice/group). Statistical analysis was performed using the Paired t-test. *P < 0.05. **(F)** RNA-sequencing data comparing healthy B and CLL cells (accession numbers GSE66117 and GSE70830) analyzed to determine differentially expressed genes; fold-changes of genes from that comparison were plotted against fold-changes when comparing peritoneal CLL cells from TCL1-Tg and TCL1-Tg BAFF^-/-^ mice. Concordance analysis identified gene expression changes in these human datasets that were also observed when comparing peritoneal CLL cells from TCL1-Tg and TCL1-Tg BAFF^-/-^ mice. **P < 0.01.

Considering that TCL1-Tg BAFF^-/-^ mice were protected against CLL in comparison to TCL1-Tg mice, we hypothesized that peritoneal CLL cells from TCL1-Tg mice may have a transcriptomic signature reflecting the aggressiveness of these cells, and that this signature should be attenuated in CLL cells from TCL1-Tg BAFF^-/-^ mice sequestered in the peritoneal cavity. Indeed, RNA-seq analysis of FACS-sorted peritoneal CLL cells isolated from either TCL1-Tg or TCL1-Tg BAFF^-/-^ mice showed differences in gene expression, which we ranked using the moderated t-statistics from a limma analysis (27) (**Figure 6A**), and by a supervised clustering approach involving a Partial Least Squares Discriminant Analysis (PLSDA) and sparse PLSDA (sPLSDA) (28) (**Figure 6B**). We further identified four top ranking up-regulated genes in peritoneal CLL cells from TCL1-Tg mice that are strongly associated with tumor progression, such as *Cd52* (**Figure 6C**). Soluble (s) CD52 is an indicator of disease activity in patients with CLL and a target of the approved drug alemtuzumab (29). The expression of genes associated with tumor promotion such as *Snap29* (30), *Mcoln1* (31) and *Nek6* (32) was up-regulated in peritoneal CLL cells from TCL1-Tg mice compared to CLL cells from TCL1-Tg BAFF^-/-^ mice (**Figure 6C**). Peritoneal CLL cells from TCL1-Tg BAFF^-/-^ mice were characterized by a tumor-suppressive gene signature with strong expression of genes such as *Prdm4* (33) and *Vezt* (34) (**Figure 6D**). Enrichment of gene sets analyses revealed that BAFF-deficient CLL cells had a significant attenuation of gene signatures associated with cellular metabolism, including oxidative phosphorylation, pyruvate metabolism, and increased glycolysis/gluconeogenesis (**Supplementary Figure 5A**). Collectively, the results suggest that peritoneal CLL cells do not require BAFF for cell survival (**Figure 3A**) but require BAFF signaling to activate a tumor-promoting gene program and a microenvironment allowing CLL progression. Indeed, we did not detect any significantly differentially expressed survival genes as part of this analysis. In contrast, BAFF deficiency is linked to up-regulation of tumor-suppressing genes in peritoneal BAFF-deficient CLL cells, which might have played a role in preventing leukemia progression to additional sites.

To validate the role of BAFF in inducing the expression of the tumor-promoting genes, peritoneal CLL cells were sorted from CLL-protected TCL1-Tg x BAFF^-/-^ mice and stimulated with BAFF *in vitro*. BAFF stimulation induced the expression of *Cd52, Mcoln1, and Snap29* in peritoneal CLL cells from TCL1-Tg BAFF^-/-^ mice (**Figure 6E**). Of note, BAFF stimulation did not affect peritoneal CLL cell numbers compared to control media alone (**Supplementary Figures 5B, C**).

We next analyzed published RNA-sequencing data and determined genes differentially expressed between human CLL B cells and healthy B cells (accession numbers GSE66117 and GSE70830) (35, 36). This data was compared with the differentially expressed genes obtained by comparing peritoneal CLL cells from TCL1-Tg and TCL1-Tg BAFF^-/-^ mice. The analysis is indicative only in the absence of any data on human B1 B cells or human CLL cells in other sites than the blood. There was a strong concordance between the gene expression changes in human and mouse studies (**Figure 6F**). We are particularly interested in genes downregulated in both human normal B cells and TCL1-Tg BAFF^-/-^ mice CLL cells relative to respective CLL counterparts (**Figure 6F**). This downregulation suggests that these genes might play a role in CLL progression and pathogenesis. Indeed, several of these common genes, including *EPB41L2* (37), *CDK6* (38)*, IL-15* (39)*, ADARB1* (40), *GCNT1* (41)*, ILDR1* (42)*, ITGA4* (43) *and TSPAN3* (44), have been reported to be associated with tumor progression. Interestingly, *in vitro* experiments demonstrated that recombinant human BAFF upregulated *EPB41L2, CDK6* and *IL15* expression in CLL cells from 5 individual patients with CLL (**Supplementary Figure 5D** and **Supplementary Table 1**), while the expression of the remaining genes did not change following BAFF stimulation, suggesting that BAFF does not directly regulate those tumor-promoting genes (**Supplementary Figure 5D**).

## Discussion

Current therapies have been designed to reduce CLL tumor burden, with significant progress made in controlling more aggressive forms of CLL (1). However, to date, no therapy is available to stop cell transformation into aggressive CLL cells. This study investigated the signals required for the initiation of CLL. We have demonstrated for the first time that BAFF signaling via its receptor BAFF-R on CLL cells is essential for the transition of peritoneal CLL cells into aggressive disseminating CLL cells but dispensable for the expansion and survival of these cells within the peritoneal cavity of TCL1-Tg mice. Peritoneal precursors of CLL in TCL1-Tg mice resemble B1 B cells in healthy mice, which also express CD5 and CD19 and do not require BAFF for development and survival (45). Our work demonstrates that in the absence of BAFF signaling, expression of the TCL1 transgene alone is insufficient for the transformation of peritoneal CLL cells into aggressive disseminating tumor cells. BAFF is known to play a dominant role on the survival of these CLL cells once these have reached peripheral tissues (5, 6), unlike in the peritoneal cavity where unidentified alternative survival mechanisms for CLL cells are at play.

Observations in the periphery are very different. The three receptors for BAFF - BAFF-R, TACI, and BCMA, are expressed on normal B cells and CLL cells (16, 22), although expression levels of these receptors may vary from patient to patient in CLL. Differences in the expression of BAFF receptors may be explained by the levels of synergistic CD40 and IL-4 signals known to up-regulate TACI expression and improve CLL cell survival in response to both BAFF and APRIL (21). Interestingly, unlike normal B cells in which BAFF-mediated survival depends on the activation of the alternate NF-κB2 pathway via BAFF-R (16), CLL cells rely on the activation of the classical NF-κB1 pathway via TACI and BCMA, as blocking BAFF-R alone did not affect CLL cell survival *in vitro* (22). Transformed and disseminated CLL cells in the periphery rely on both BAFF and APRIL for survival and accumulation, with a possible vital role for the receptor TACI. Indeed, we demonstrated that TACI signaling is responsible for IL-10 production by CLL cells (46). IL-10 is an immunosuppressive cytokine that may contribute to immune evasion of CLL cells and facilitate CLL expansion. Our work showed a dominant role for BAFF in supporting CLL initiation and expansion in the periphery with APRIL playing no additive role.

Ibrutinib blocks Bruton’s tyrosine kinase (BTK) downstream of the BCR. BTK signaling is required for TACI expression, linking the mechanism of action of Ibrutinib to a reduction of TACI expression (47). Similarly, Venetoclax is an inhibitor of the pro-survival oncogene Bcl-2, the expression of which is increased in response to BAFF signaling (48). Idelalisib is an inhibitor of the p110d catalytic site of PI3K, required for BAFF-mediated B cell survival (48). Therefore, combining BAFF inhibition with either Ibrutinib, Idelalisib or Venetoclax would not be expected to show any significant added benefits, as the targeted pathways are interconnected (48). Yet observations have been different. Indeed, the anti-BAFF-R blocking antibody VAY-736 enhances the effect of ibrutinib in TCL1-Tg mice (49), supporting the notion that BAFF signaling is not merely restricted to a cell survival function. The BAFF-BAFF-R axis was shown to mediate the initiation and dissemination of CLL in this study, possibly via up-regulation of tumor-promoting genes. Therefore, it is possible that combining VAY-736 and Ibrutinib may have inhibited both the output of disseminating CLL cells from the peritoneal cavity (VAY-736) and the survival of existing transformed CLL cells (VAY-736 and Ibrutinib) in the periphery, thus showing greater efficacy than their use in isolation.

The expression of *Cd52*, *Snap29, Mcoln1 and Nek6* genes, known to promote tumorigenesis in different cancer types, was up-regulated in peritoneal TCL1-Tg CLL cells. CLL cells express surface CD52, and elevated levels of serum CD52 have been detected in patients with progressive CLL (29). Alemtuzumab (CAMPATH-1H), an anti-CD52 antibody, has been approved for treating refractory CLL (50). Our study is the first to reveal that BAFF is necessary for high CD52 expression in CLL cells, as evidenced by BAFF signaling directly upregulating *Cd52* expression in CLL cells upon stimulation *in vitro*. Expression of several additional factors is elevated in TCL1-Tg CLL cells, such as Snap29 involved in cellular trafficking and autophagy (30). Inhibition of Snap29 enhances apoptosis and inhibits autophagy in cancer cells (51). Another factor is Mcoln1 which regulates oncogenic autophagy in different solid cancers (52). Its inhibition suppresses tumor cell proliferation and growth *in-vitro* and *in-vivo* (31). The tumor-suppressor p53 is known to repress Mcoln1, inhibiting cancer cell proliferation and invasion (53). Mutation or inactivation of *p53*, as observed in CLL (54), augments Mcoln1 abundance, which fuels cancer progression (53). *Never in Mitosis gene A* (NIMA)-related *kinase 6* (Nek6) was initially discovered as a regulator of mitotic cell division and inhibition of Nek6 results in apoptosis (55). Nek6 is highly expressed in different malignant tumors, and elevated expression correlates with tumor stage and poor prognosis (56, 57). We observed reduced *Nek6* expression in peritoneal TCL1-Tg BAFF^-/-^ CLL cells, suggesting reduced cell cycle progression and possibly explaining reduced numbers of these cells in the peritoneal cavity TCL1-Tg BAFF^-/-^ mice compared to the same site in TCL1-Tg controls.

In contrast, genes strongly expressed in peritoneal TCL1-Tg BAFF^-/-^ CLL cells are associated with anti-tumor functions. Upregulation of tumor-suppressing genes *Prdm4* and *Vezt* was also observed in these cells. *Prdm4* is a transcription regulator located in the tumor suppressor locus involved in cell proliferation and differentiation (58). Prdm4 inactivates PI3K/AKT signaling (also promoted by TCL1), which inhibits cancer cell proliferation and tumor formation (33). Vezt is a component of adherens junctions in the plasma membrane, and its expression is downregulated in gastric cancer tissues (34). Importantly, inhibition of *Vetz* promoter methylation increases *Vezt* expression, leading to cell cycle arrest and tumor suppression (34).

Our observation that the gene profile of peritoneal CLL cells in protected TCL1-Tg BAFF^-/-^ mice shared more similarities with that of healthy human B cells supported some clinical relevance of our pre-clinical study. Some of the genes which expression was downregulated in both TCL1-Tg BAFF^-/-^ CLL cells and healthy human B cells, were upregulated in human CLL cells upon BAFF stimulation in culture, in particular *IL-15* which plays a known role in CLL progression (39). This comparison is of course only indicative of the direct role of BAFF on the expression of some relevant human CLL genes but is very much limited by the fact that no data on human peritoneal CLL or B1 cell is available. The peritoneum is not a site routinely checked for residual CLL disease in the clinic. In addition, identification of human B1 cells has been difficult, debated but with recent data suggesting that this subset may exist and present in very low numbers in healthy individuals (59). A number of studies suggest that human B1 (60) and a similar subset of IL-10-producting regulatory B cells (Breg) (61) may be normal counterparts of human CLL cells. Interestingly, similar to observation with mouse B1 B cells, human B1 B cells appear to be minimally reliant on BAFF for survival and resisted treatment with the BAFF inhibitor belimumab in patients with systemic lupus erythematosus (62). More work is required to determine whether human B1 B cells may be relevant as a target precursor B cell subset in human CLL.

In conclusion, our work has unveiled an unappreciated role for BAFF in experimental CLL progression. BAFF is not needed for the survival and expansion of CLL cells in the peritoneal cavity of TCL1-Tg mice. Instead, BAFF binding to BAFF-R activates a tumor-promoting gene program in the peritoneal CLL cells, potentially allowing the progression of CLL. Our results suggest that blocking the BAFF-BAFF-R axis in combination with existing therapies could potentially achieve two goals; reduce tumor burden in the periphery and eliminate the supply of more aggressive precursor CLL cells.

## Data availability statement

The data presented in the study are deposited in the NCBI BioProject database (https://www.ncbi.nlm.nih.gov/bioproject/) repository, accession number PRJNA1054221.

## Ethics statement

The studies involving humans were approved by the Royal Brisbane & Women’s Hospital Human Research Ethics Committee. The studies were conducted in accordance with the local legislation and institutional requirements. The participants provided their written informed consent to participate in this study. The animal study was approved by the QIMR Berghofer Medical Research Institute Animal Research Ethics Committee. The study was conducted in accordance with the local legislation and institutional requirements.

## Author contributions

MU: Data curation, Project administration, Writing – original draft. BG: Data curation, Investigation, Methodology, Project administration, Writing – original draft. EW: Data curation, Formal analysis, Methodology, Validation, Writing – review & editing. WF: Formal analysis, Writing – review & editing. YS: Data curation, Formal analysis, Writing – review & editing. SI: Data curation, Formal analysis, Methodology, Writing – review & editing. ME: Data curation, Formal analysis, Writing – review & editing. SY: Data curation, Formal analysis, Writing – review & editing. MR: Formal analysis, Writing – review & editing. NW: Data curation, Resources, Writing – review & editing. PS: Data curation, Resources, Supervision, Writing – review & editing. CT: Supervision, Validation, Writing – review & editing. FM: Conceptualization, Formal analysis, Funding acquisition, Investigation, Project administration, Resources, Supervision, Validation, Writing – original draft.
