# Supplementary material for: An unappreciated cell survival-independent role for BAFF initiating chronic lymphocytic leukemia
Source: Front Immunol. 2024 Feb 26;15:1345515. doi: 10.3389/fimmu.2024.1345515 (PMC10927009; doi:10.3389/fimmu.2024.1345515)
Supplement: Supplementary file 1 [file DataSheet_1.pdf]

Supplemental text

MATERIALS AND METHODS

Flow cytometric analysis

Immune cells from blood, peritoneal lavage, spleen, lymph nodes, bone marrow (BM) and omentum were harvested. Peritoneal lavages were centrifuged, cells washed and prepared into single-cell suspensions. Spleen, lymph nodes and omentum were disaggregated mechanically against a 70 µM cell strainer (Corning Inc, MA, USA) to prepare single-cell suspensions. Femur bones were isolated and cleaned, and BM cells were flushed out using FACS buffer (2% fetal calf serum and 2mM EDTA in PBS)-loaded syringe fitted with a 25 gauge needle and further passed through a 70 µM cell strainer. With BM and spleen samples, red blood cells (RBC) were lysed with RBC lysis buffer (eBiosciences™, CA, USA) before cells were washed and resuspended in FACS buffer. Cells were stained with Zombie Aqua™ Fixable Viability Kit (Biolegend, CA, USA) for 15 minutes at room temperature. Cells were washed with FACS buffer and further incubated with Fc block antibody (2.4G2; BD Biosciences, CA, USA) for 30 minutes at 4^0C to block non-specific Fc binding. After washing with FACS buffer, cells were stained with fluorochrome-conjugated antibodies for 30 minutes at 4^0C. Depending on the experiment (detailed in figure legends), different cocktails of the following fluorescence-labeled anti-mouse monoclonal antibodies were used: CD45.2 – BV711 (104), CXCR4 – PE (2B11) and CXCR5 – PE Cy7 (2G8) from BD Biosciences (CA, USA); CD19 - PE Cy7, FITC (6D5) and CD5 – APC (53-7.3) from Biolegend (CA, USA); BAFF-R – FITC (eBio7H22-E16), CD9 – eFluor (KMC8) and TACI – PE (ebio8F10-3) from eBiosciences™ (CA, USA); BCMA – APC-Vio770 (REA550) from Miltenyi Biotech (Germany). Blood samples were stained at room temperature for 30 min with fluorochrome-conjugated antibodies and then RBC lysed with RBC lysis buffer before analysis. Absolute cell numbers in all samples were

enumerated using CountBright™ absolute counting beads (ThermoFisher Scientific, MA, USA). Data were acquired using a BD Fortessa or BD LSR-II flow cytometer (BD Biosciences, CA, USA) and analyzed with FlowJo software (version 10.7; TreeStar, OR, USA).

CLL cell culture and gene expression analysis

Peritoneal CLL cells were FACS-sorted from peritoneal lavage of TCL1-Tg and TCL1-Tg BAFF$^{-/-}$ mice (8 to 9 months old). The cells were seeded at 1 x 10^5 cells per well in RPMI-1640 media (Gibco, Thermo Fisher, MS, USA) supplemented with FCS (10%; Thermo Fisher, MS, USA), sodium pyruvate (1mM; Gibco, Thermo Fisher, MS, USA), L-glutamine (2mM; 1mM; Gibco, Thermo Fisher, MS, USA), HEPES (20mM; 1mM; Gibco, Thermo Fisher, MS, USA), Penicillin-Streptomycin (100U/ml; Gibco, Thermo Fisher, MS, USA), and 2-Mercaptoethanol (50μM; Sigma-Aldrich, MO, USA). Cells were stimulated with media only or 50 ng/ml of recombinant mouse BAFF (R&D Systems, MN, USA). Human CLL cells were magnetically sorted from the peripheral blood mononuclear cells (PBMC) of CLL patients using a human B-CLL cell isolation kit according to the manufacturer's instructions (Miltenyi Biotec, Germany). Patients's details are provided in Supplemental Table 1. CLL cells were seeded at 1 x 10^5 cells per well in supplemented RPMI-1640 media (as above) and stimulated with media or 50 ng/ml of recombinant human BAFF (Adipogen Corporation, CA, USA). Twenty-four hours later, cells were harvested. The number of cells was enumerated using CountBright™ absolute counting beads, and viability was tested using PE Annexin V Apoptosis Detection Kit with 7-AAD (Biolegend, CA, USA). Additional cells were stored in TRIzol™ Reagent (Invitrogen, Thermo Fisher, MS, USA) at -80^{0}C for RNA extraction. RNA was extracted as per the manufacturer's protocol. A reverse transcription polymerase chain reaction (PCR) was performed using a High-Capacity cDNA Reverse Transcription Kit (Applied Biosystems, Thermo Fisher Scientific corporation, MS, USA) to generate complementary DNA (cDNA).

An RT-qPCR was run using PowerUp™ SYBR™ Green Master Mix (Applied Biosystems, Thermo Fisher Scientific corporation, MS, USA) using the following primers (1):

Mouse *HPRT*: Forward 5’-GTCCCAGCGTCGTGATTAG-3’

Reverse 5’-TTTCCAAATCCTCGGCATAATGA-3’

Mouse *CD52*: Forward 5’-ATCCTTGGGACAAGCCACTAC-3’

Reverse 5’-GGCACATTAAGGTATTGGCAAAG-3’

Mouse *Mcoln1*: Forward 5’-CTGACCCCCAATCCTGGGTAT-3’

Reverse 5’-GGCCCGGAACTTGTCACAT-3’

Mouse *Snap29*: Forward 5’-TTCGACGATGACGTGGAAGAG-3’

Reverse 5’-GGTACTGCTGCCTGTCAATGG-3’

Mouse *Nek6*: Forward 5’-CCCTGTCTTTTCGCTGCTCA-3’

Reverse 5’-CAGGCAAGTGGCCTTGTAAA-3’

Human *EPB41L2*: Forward 5’- GTGCTTAAAAGCTGCCATAAG -3’

Reverse 5’- CCTTGGTTGCATCTGTTCCT-3’

Human *CDK6*: Forward 5’- CCCTGCAACCTCTCCGC -3’

Reverse 5’- TCAGTCCAGAATCATTGCACTTTT-3’

Human *IL15*: Forward 5’- AGTTGGCCCAAAGCACCTAA -3’

Reverse 5’- GCACTGAAACAGCTGCACAA-3’

Human *ADARB1*: Forward 5’- TTTTCAGCATCATGGGGCG -3’

Reverse 5’- AGTGCTGCTGGAACTCATGT -3’

Human *GCNT1*: Forward 5’- CAGAATGGGGCAGGATGTCA -3’

Reverse 5’- TCTTGAAATGAAGAGCAGCACATT -3’

Human *ILDR1*: Forward 5’- ACCATCCAGAACCCCCTGG -3’

Reverse 5’- GAAACCTGGGAGCTCCTGTC -3’

Human *ITGA4*: Forward 5’- GATGCTGTTGCTGTGCCTG -3’

Reverse 5'- CCCACTAGGAGCCATCGGTT -3'

Human *TSPAN3*: Forward 5'- ACCTCATCTTCTGGTTTGTCAT -3'

Reverse 5'- GAATGCTGCGATCAACCTCA -3'

The expression of each target gene was normalized to the housekeeping gene *HPRT* and expressed as fold change over paired unstimulated samples using the $2^{-\Delta\Delta Ct}$ formula.

RNA extraction and RNA-sequencing

Peritoneal CLL cells were FACS-sorted (>94% purity) from peritoneal lavage of TCL1-Tg and TCL1-Tg BAFF$^{-/-}$ mice (8 to 9 months old). RNA was extracted from these cells using an RNeasy Kit (QIAGEN, Germany). RNA libraries were prepared for RNA-sequencing (RNA-seq) using standard Illumina protocols. RNA-seq was performed on an Illumina NovaSeq platform (Illumina, California, USA), with HiSeq Control Software (HCS) v2.2.68 and Real-Time Analysis (RTA) v1.18.66.3 running on the instrument computer. 100 bp single-end reads were analyzed with the bcl2fastq 2.20.0.422 pipeline. The RNA-seq data have been deposited with links to BioProject accession number PRJNA1054221 in the NCBI BioProject database (https://www.ncbi.nlm.nih.gov/bioproject/).

Read quality, trimming, mapping, and summarization

The ends of the reads were trimmed with Trimmomatic (v0.39) using a sliding window quality filter (2). Reads were mapped using HISAT2 (3) (v2.2.1) to the Mus musculus reference genome (GRCm39 assembly, Ensembl release-106) and matched to the GENCODE Release M20 gene annotation. Read counts were summarized using the *featureCounts* function of the Subread software package (v2.0.2) (4); non-uniquely mapped reads (i.e., reads that map to more than one gene ambiguously) were intentionally excluded from the analysis. While males

and females were included, Y-chromosome genes were excluded from the analyses. Genes with low expression were filtered out using a threshold requiring at least 2 counts per million (cpm) in half of the samples. Public human RNAseq datasets (GSE66117 and GSE70830) were similarly processed using the human reference genome (GRCh38).

Count-based expression analyses, gene selection and clustering

Read counts were normalized by the upper-quartile method to correct differences in sequencing depth between samples (5). The limma/edgeR workflow was used for differential expression analysis (6). Expression profiles of selected genes were viewed as a heatmap using ComplexHeatmap (7). Transcriptomes were analysed by partial least squares discriminant analysis (PLSDA) and sparse PLSDA (sPLSDA) using the MixOmics R package (v6.20.0) (8); genes with predictive values were selected using cross-validation to protect against overfitting using M-fold cross-validation (10-folds averaged 50 times). Log2 fold-changes comparing TCL1-Tg BAFF$^{-/-}$ CLL cells to TCL1-Tg CLL cells were plotted against the log2 fold-changes from public human datasets (GSE66117 and GSE70830) comparing healthy donor B cells to CLL cells using the ggplot2 package (9), after filtering out non-homologous human/mouse gene symbols. Plot symbols represent genes, with a color scale indicating the mean of autoscaled fold-changes from each comparison.

Gene set enrichment tests

The EGSEA (v1.24.0) R package was used to statistically test for enrichment of gene expression signatures at a systems level, derived from an ensemble of several gene set enrichment analyses tools (10). EGSEA uses count data transformed with *voom* (a function of the limma package) (11). Collections of pre-defined gene set collections were used: KEGG Pathways, and the Broad Institute MSigDB collections (12).

REFERENCES

1. Wang X, et al. PrimerBank: a PCR primer database for quantitative gene expression analysis, 2012 update. *Nucleic acids research.* 2011;40(D1):D1144-D1149.
2. Bolger AM, et al. Trimmomatic: a flexible trimmer for Illumina sequence data. *Bioinformatics.* 2014;30(15):2114-2120.
3. Pertea M, et al. Transcript-level expression analysis of RNA-seq experiments with HISAT, StringTie and Ballgown. *Nat Protoc.* 2016;11(9):1650-1667.
4. Liao Y, et al. The Subread aligner: fast, accurate and scalable read mapping by seed-and-vote. *Nucleic acids research.* 2013;41(10):e108.
5. Bullard JH, et al. Evaluation of statistical methods for normalization and differential expression in mRNA-Seq experiments. *BMC Bioinformatics.* 2010;11:94.
6. Law CW, et al. RNA-seq analysis is easy as 1-2-3 with limma, Glimma and edgeR. *F1000Res.* 2016;5:1408.
7. Gu Z, et al. Complex heatmaps reveal patterns and correlations in multidimensional genomic data. *Bioinformatics.* 2016;32(18):2847-2849.
8. Le Cao KA, et al. Sparse PLS discriminant analysis: biologically relevant feature selection and graphical displays for multiclass problems. *BMC Bioinformatics.* 2011;12:253.
9. H W. *ggplot2: Elegant Graphics for Data Analysis.* Springer-Verlag New York; 2016.
10. Alhamdoosh M, et al. Combining multiple tools outperforms individual methods in gene set enrichment analyses. *Bioinformatics.* 2017;33(3):414-424.
11. Law CW, et al. voom: Precision weights unlock linear model analysis tools for RNA-seq read counts. *Genome Biol.* 2014;15(2):R29.
12. Subramanian A, et al. Gene set enrichment analysis: a knowledge-based approach for interpreting genome-wide expression profiles. *Proc Natl Acad Sci U S A.* 2005;102(43):15545-15550.

SUPPLEMENTAL FIGURE LEGENDS

Supplemental Figure 1. Reduced blood CLL cell number and CLL incidence in TCL1-Tg -BAFF$^{-/-}$ mice.

(A) Absolute numbers of CD19$^+$CD5$^+$ cells per ml of blood of 2 to 12 month old TCL1-Tg, TCL1-Tg BAFF$^{-/-}$ and TCL1-Tg APRIL$^{-/-}$ mice determined by flow cytometry. Data are presented as mean values ± SEM. Differences between the groups were determined using two-way ANOVA with Bonferroni post hoc test. (**B)** Kaplan-Meier curve depicting overall CLL incidence, determined as >10^4 CLL cells/µl of blood, from birth to ethical endpoint. Statistical analysis was performed using the Log-rank (Mantel-Cox) test. Not significant (ns), ****P < 0.0001.

Supplemental Figure 2. BAFF, but not APRIL, is required for CLL initiation and progression in TCL1-Tg mice.

(A) Absolute numbers of CD19$^+$CD5$^+$ cells in the peritoneal cavity, spleen, blood (per 1 µl), bone marrow and lymph nodes of 6 to >10-month old TCL1-Tg, TCL1-Tg BAFF$^{-/-}$, TCL1-Tg APRIL$^{-/-}$, WT, BAFF$^{-/-}$ and APRIL$^{-/-}$ mice, as indicated. (**B)** The absolute number of CD19$^+$CD5$^+$ cells in the peritoneal cavity, spleen, blood (per 1 µl), bone marrow and lymph nodes of >10 month old TCL1-Tg, TCL1-Tg BAFF-R$^{-/-}$, WT and BAFF-R$^{-/-}$ mice. Statistically significant differences were calculated using two-way ANOVA with Bonferroni post hoc test. Not significant (ns), *P < 0.05, **P < 0.01, ***P < 0.001.

Supplemental Figure 3 . CLL cells primarily accumulate in the peritoneal cavity but not in the omentum in TCL1-Tg BAFF$^{-/-}$ mice.

Percentages of CD19$^+$CD5$^+$ lymphocytes in the peritoneal cavity lavage (PerC) and omentum of 8 month old TCL1-Tg BAFF$^{-/-}$ mice as indicated.

Supplementary Figure 4 . Treatment with anti-APRIL antibody does not prevent expansion or sequestration of CLL cells in the peritoneal cavity of TCL1-Tg BAFF$^{-/-}$ mice.

4-5 mo old TCL1-Tg and TCL1-Tg BAFF$^{-/-}$ mice were injected i.p. twice weekly with a neutralizing anti-APRIL antibody or isotype control antibody for 8 weeks. (**A)** The percentage of CD19$^+$CD5$^+$ cells and (**B)** the absolute number of CD19$^+$CD5$^+$ cells in the peritoneal cavity were determined 7 days post last treatment. Statistically significant

differences were calculated using one-way ANOVA with Bonferroni post hoc test. Not significant (ns).

Supplemental Figure 5. Separate gene profile TCL1-Tg BAFF$^{-/-}$ peritoneal CLL cells sharing similarities with human B cells.

A Gene sets enrichment analysis for the oxidative phosphorylation, pyruvate metabolism and glycolysis/gluconeogenesis pathways in peritoneal CLL cells of TCL1-Tg and TCL1-Tg BAFF$^{-/-}$ mice. Vertical bars mark genes which belong to gene sets distributed among all genes ranked according to a moderated t-statistic, comparing the two experimental groups. **B** Absolute cell numbers and **C** proportions of dead cells (n = 7 mice) in 24h cultures with purified peritoneal CLL cells from TCL1-Tg BAFF$^{-/-}$ mice stimulated or not with recombinant mouse BAFF. **D** Human CLL cells sorted from PBMC and stimulated or not 24 hours with recombinant human BAFF (n = 5 patients). Gene expression (as indicated) was assessed by qPCR. Statistical analysis was performed using the Mann-Whitney t-test. **P < 0.01. **E** Mechanism of BAFF-BAFF-R-mediated CLL transformation in the peritoneal cavity. BAFF-BAFF-R signaling upregulates tumor-promoting genes in peritoneal CD19^{+}CD5^{+} TCL1-Tg B cells, resulting in the leukemic dissemination of CLL cells in the periphery. In the absence of BAFF, CD19^{+}CD5^{+} TCL1-Tg B cells upregulate tumor-suppressing genes, and most cells remain in the peritoneal cavity.

SUPPLEMENTAL TABLE.

Supplemental Table 1: CLL patients' details

ID	CLL-1	CLL-2	CLL-3	CLL-4	CLL-5
**Age**	68	45	66	63	76
**Sex**	Male	Male	Male	Male	Male
**Rai Stage**	4	1	2	4	2
**Treatment status**	Untreated	Untreated	Untreated	Untreated	Untreated
**% CLL in blood**	10.4%	44.5%	75.5%	64.3%	77.7%

Supplemental Table 2: Spleen of the mice in Figure 2D

Spleen weights (g) 6-7 months		
WT	BAFF -/-	APRIL -/-
0.13	0.07	0.13
0.09	0.05	0.12
0.15	0.07	0.15
0.09	0.059	0.112
0.09	0.056	0.098
0.08	0.043	
0.16		
0.11		
0.14		

Spleen weights (g) 8-9 months		
WT	BAFF -/-	APRIL -/-
0.12	0.041	0.106
0.14	0.083	0.157
0.11	0.11	0.109
0.11	0.08	0.095
0.11	0.08	0.157
0.18		0.109
0.11		0.095
0.14		
0.13		

Spleen weights (g) >10 months		
WT	BAFF -/-	APRIL -/-
0.1	0.05	0.115
0.125	0.043	0.148
0.12	0.056	0.114
0.09	0.054	
0.11	0.095	
0.12	0.041	
0.13		
0.1		
0.12		
0.11		
0.09		
0.1		
0.13		
0.17		
0.12		

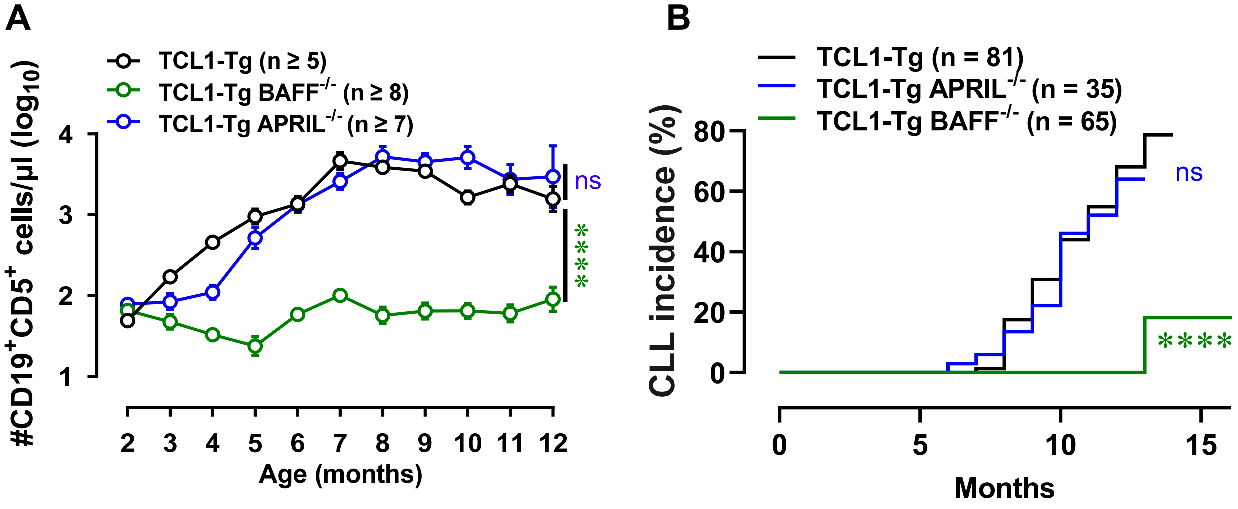

Supplementary Figure 1. Reduced blood CLL cell number and CLL incidence in TCL1-Tg -BAFF$^{-/-}$ mice.

(A) Absolute numbers of CD19$^+$CD5$^+$ cells per µl of blood of 2 to 12 mo old TCL1-Tg, TCL1-Tg BAFF$^{-/-}$ and TCL1-Tg APRIL$^{-/-}$ mice determined by flow cytometry. Data are presented as mean values ± SEM. Differences between the groups were determined using two-way ANOVA with Bonferroni post hoc test. (**B)** Kaplan-Meier curve depicting overall CLL incidence, determined as >10^4 CLL cells/µl of blood, from birth to ethical endpoint. Statistical analysis was performed using the Log-rank (Mantel-Cox) test. Not significant (ns), ****P < 0.0001.

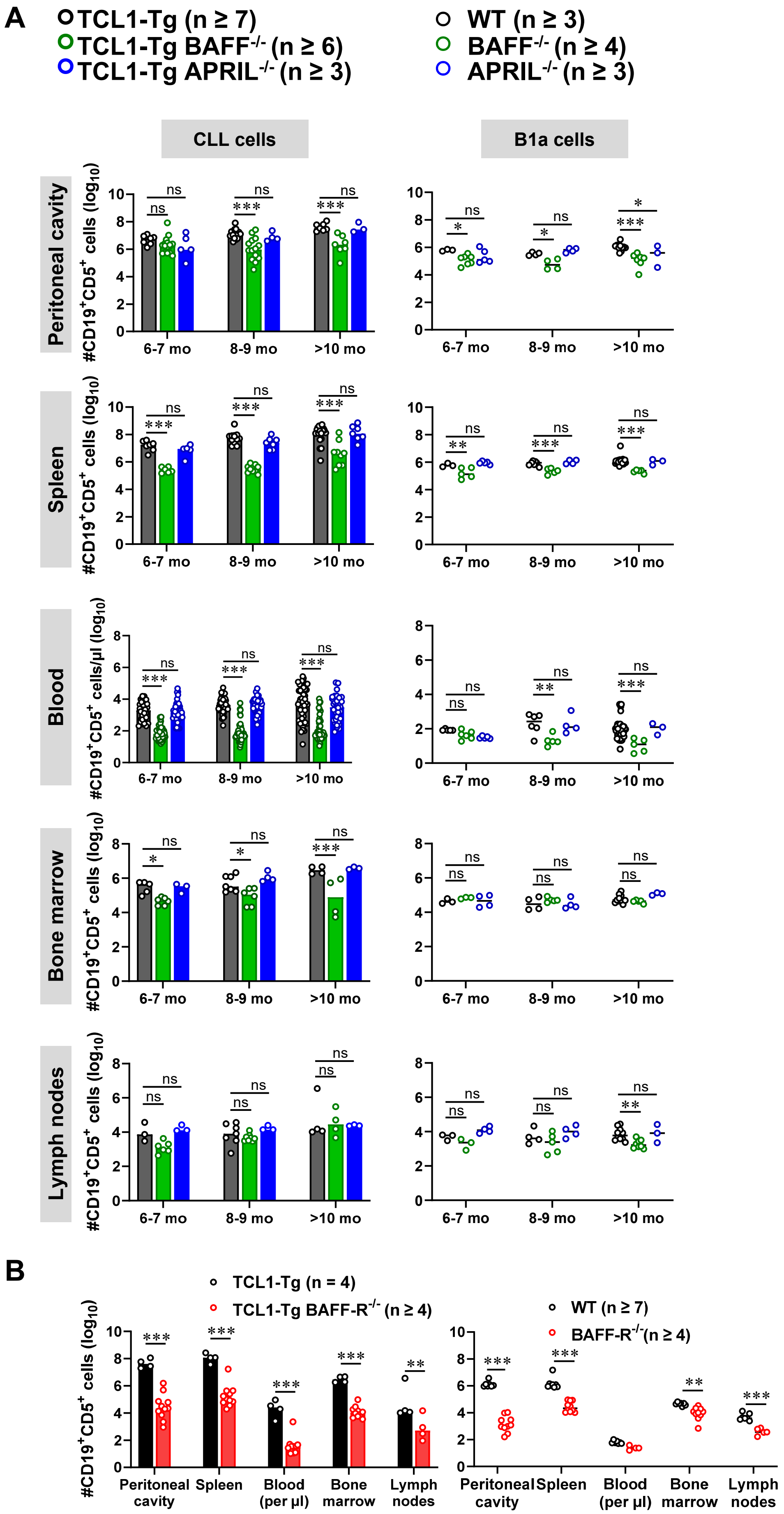

A
TCL1-Tg (n ≥ 7)
TCL1-Tg BAFF-/- (n ≥ 6)
TCL1-Tg APRIL-/- (n ≥ 3)
WT (n ≥ 3)
BAFF-/- (n ≥ 4)
APRIL-/- (n ≥ 3)
CLL cells
B1a cells
Peritoneal cavity
Spleen
Blood
Bone marrow
Lymph nodes
#CD19+CD5+ cells (log10)
#CD19+CD5+ cells/µl (log10)
6-7 mo
8-9 mo
>10 mo
ns
*
**
***
B
TCL1-Tg (n = 4)
TCL1-Tg BAFF-R-/- (n ≥ 4)
WT (n ≥ 7)
BAFF-R-/-(n ≥ 4)
Peritoneal cavity
Spleen
Blood (per µl)
Bone marrow
Lymph nodes

Supplementary Figure 2. BAFF, but not APRIL, is required for CLL initiation and progression in TCL1-Tg mice.

(A) Absolute numbers of $CD19^+CD5^+$ cells in the peritoneal cavity, spleen, blood (per 1 μl), bone marrow and lymph nodes of 6 to >10-mo old TCL1-Tg, TCL1-Tg $BAFF^{-/-}$, TCL1-Tg $APRIL^{-/-}$, WT, $BAFF^{-/-}$ and $APRIL^{-/-}$ mice, as indicated. **(B)** The absolute number of $CD19^+CD5^+$ cells in the peritoneal cavity, spleen, blood (per 1 μl), bone marrow and lymph nodes of >10 mo old TCL1-Tg, TCL1-Tg BAFF-$R^{-/-}$, WT and BAFF-$R^{-/-}$ mice. Statistically significant differences were calculated using two-way ANOVA with Bonferroni post hoc test. Not significant (ns), *P < 0.05, **P < 0.01, ***P < 0.001. The percentages of cells are also depicted in Figure 3.

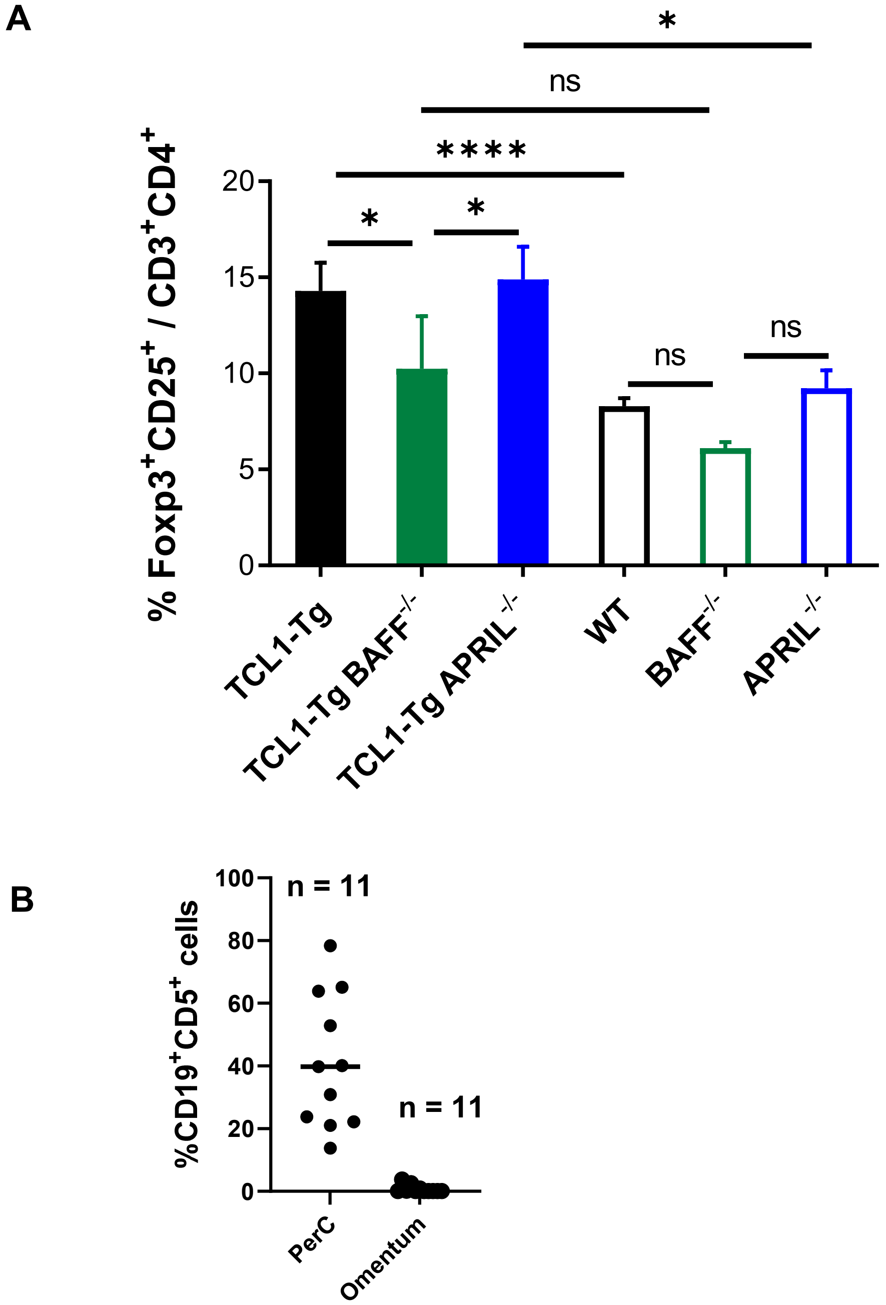

Supplementary Figure 3 . Reduced expansion of regulatory T cells (A) and predominant peritoneal cavity accumulation of CLL cells, not in the omentum (B), in TCL1-Tg BAFF$^{-/-}$ mice.

(A) Percentages of regulatory T cells (Foxp3^{+}CD25^{+}) in the spleen of 6 to 10 months old TCL1-Tg, TCL1-Tg BAFF$^{-/-}$, TCL1-Tg APRIL$^{-/-}$, WT, BAFF$^{-/-}$ and APRIL$^{-/-}$ mice determined by flow cytometry Statistical analysis was performed using the One – way ANOVA (and Nonparametric) Kruskal-Walls test for multiple comparisons(n ≥ 4). *P < 0.05 ****P < 0.0001. **(B)**. Percentages of CD19+CD5+ lymphocytes in the peritoneal cavity lavage (PerC) and omentum of 8 mo old TCL1-Tg BAFF-/- mice as indicated.

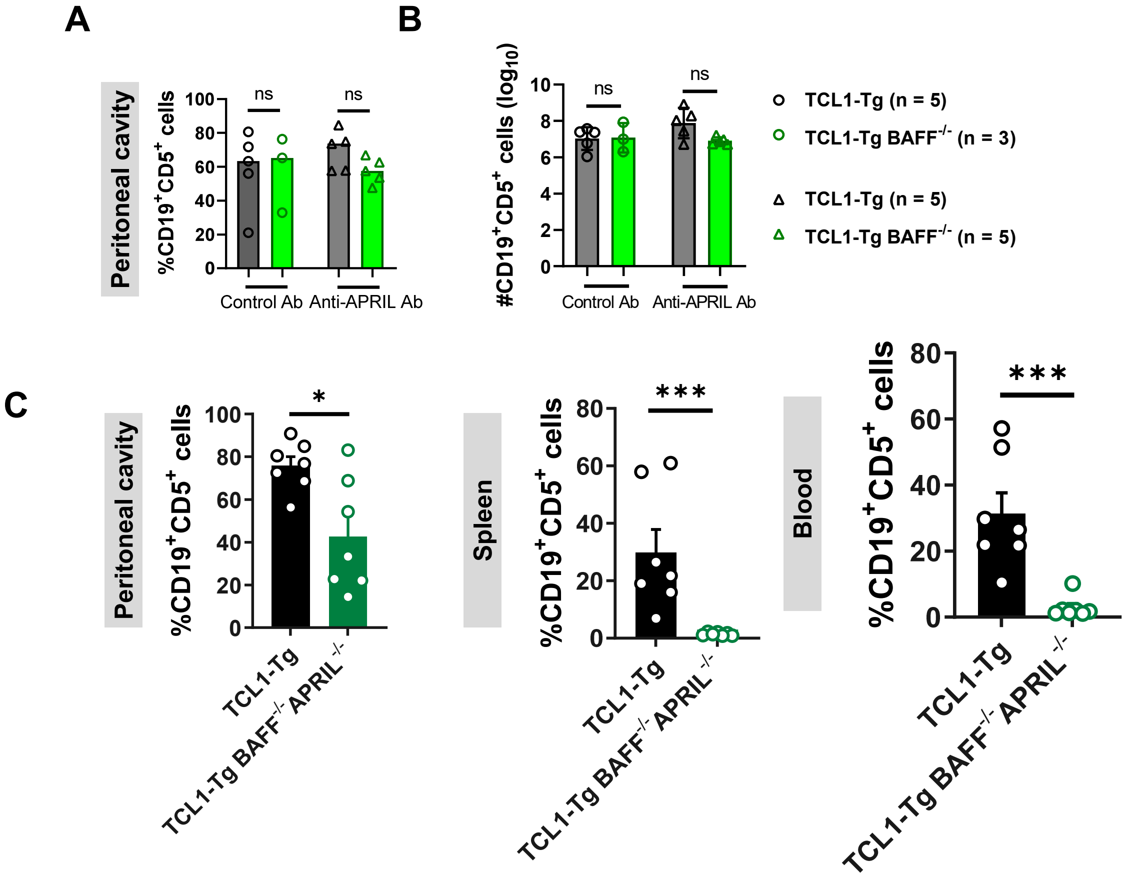

Supplementary Figure 4 . Treatment with anti-APRIL antibody or APRIL deficiency does not prevent the expansion or sequestration of CLL cells in the peritoneal cavity of TCL1-Tg BAFF$^{-/-}$ mice.

4-5 mo old TCL1-Tg and TCL1-Tg BAFF$^{-/-}$ mice were injected i.p. twice weekly with a neutralizing anti-APRIL antibody or isotype control antibody for 8 weeks. (**A)** The percentage of CD19^{+}CD5^{+} cells and (**B)** the absolute number of CD19^{+}CD5^{+} cells in the peritoneal cavity were determined 7 days post last treatment. Statistically significant differences were calculated using one-way ANOVA with Bonferroni post hoc test (A &B). Not significant (ns). **(C)** Percentages of CD19^{+}CD5^{+} cells (CD45.2^{+} live gate) in the peritoneal cavity, spleen and blood of 6-7 months old TCL1-Tg, TCL1-Tg BAFF$^{-/-}$APRIL$^{-/-}$, as indicated. Statistical analysis was performed using the Mann-Whitney t-test (n=7). *P < 0.05 ***P < 0.001.

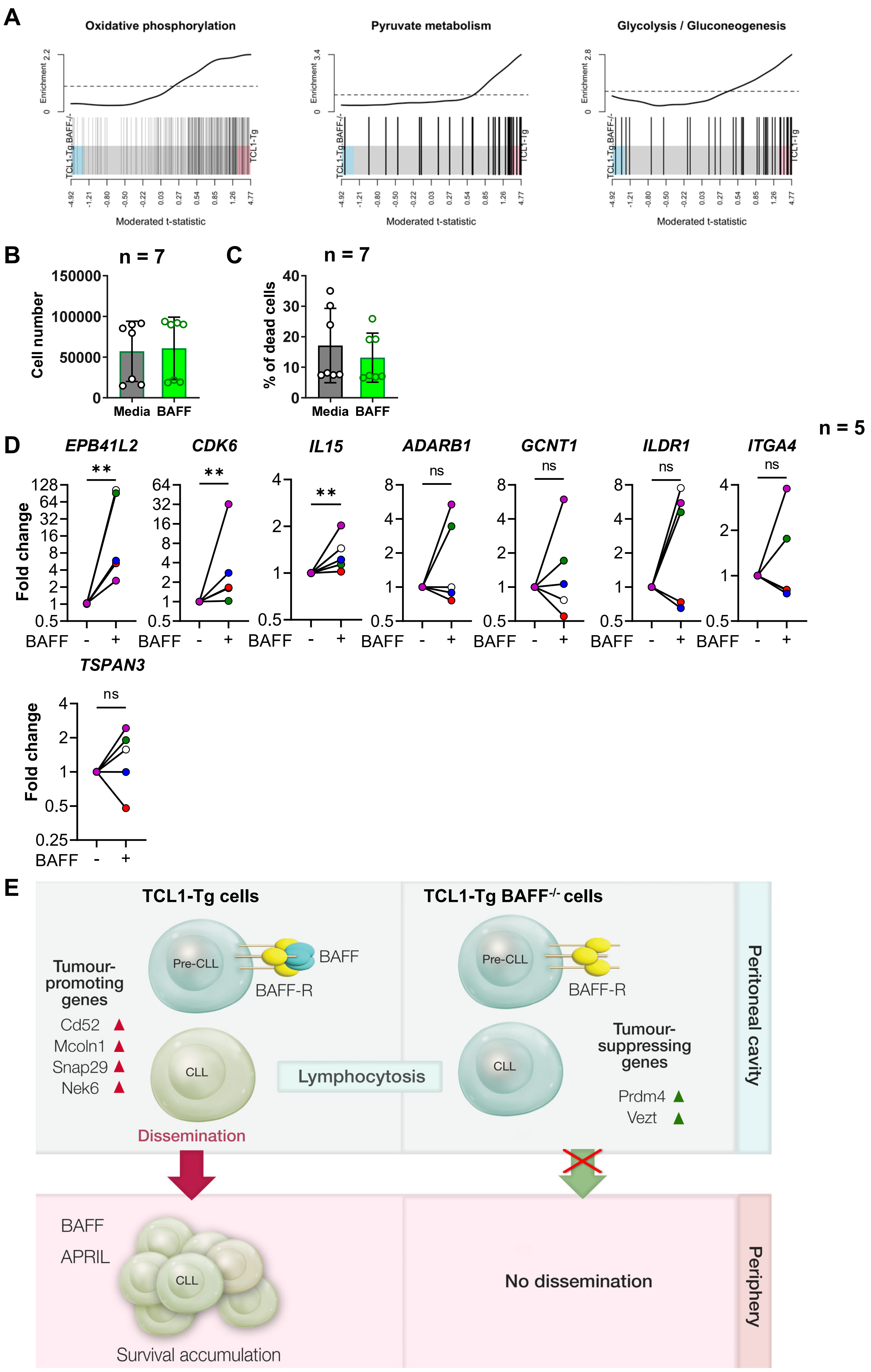

A
Oxidative phosphorylation
Pyruvate metabolism
Glycolysis / Gluconeogenesis
Enrichment
TCL1-Tg.BAFF-/-
TCL1-Tg
Moderated t-statistic
B
n = 7
Cell number
Media BAFF
C
n = 7
% of dead cells
Media BAFF
D
n = 5
EPB41L2
CDK6
IL15
ADARB1
GCNT1
ILDR1
ITGA4
TSPAN3
Fold change
BAFF
E
TCL1-Tg cells
TCL1-Tg BAFF-/- cells
Pre-CLL
BAFF
BAFF-R
Tumour-promoting genes
Cd52
Mcoln1
Snap29
Nek6
CLL
Lymphocytosis
Tumour-suppressing genes
Prdm4
Vezt
Dissemination
Peritoneal cavity
BAFF
APRIL
Survival accumulation
No dissemination
Periphery

Supplementary Figure 5. Separate gene profile TCL1-Tg BAFF$^{-/-}$ peritoneal CLL cells sharing similarities with human B cells.

A Gene sets enrichment analysis for the oxidative phosphorylation, pyruvate metabolism and glycolysis/gluconeogenesis pathways in peritoneal CLL cells of TCL1-Tg and TCL1-Tg BAFF$^{-/-}$ mice. Vertical bars mark genes which belong to gene sets distributed among all genes ranked according to a moderated t-statistic, comparing the two experimental groups. **B** Absolute cell numbers and **C** proportions of dead cells (n = 7 mice) in 24h cultures with purified peritoneal CLL cells from TCL1-Tg BAFF$^{-/-}$ mice stimulated or not with recombinant mouse BAFF. **D** Human CLL cells sorted from PBMC and stimulated or not 24 hours with recombinant human BAFF (n = 5 patients). Gene expression (as indicated) was assessed by qPCR. Statistical analysis was performed using the Mann-Whitney t-test. **P < 0.01. **E** Mechanism of BAFF-BAFF-R-mediated CLL transformation in the peritoneal cavity. BAFF-BAFF-R signaling upregulates tumor-promoting genes in peritoneal CD19^{+}CD5^{+} TCL1-Tg B cells, resulting in the leukemic dissemination of CLL cells in the periphery. In the absence of BAFF, CD19^{+}CD5^{+} TCL1-Tg B cells upregulate tumor-suppressing genes, and most cells remain in the peritoneal cavity.
